# Stillbirths: Contribution of preterm birth and size‐for‐gestational age for 125.4 million total births from nationwide records in 13 countries, 2000–2020

**DOI:** 10.1111/1471-0528.17653

**Published:** 2023-11-29

**Authors:** Yemisrach B. Okwaraji, Lorena Suárez‐Idueta, Eric O. Ohuma, Ellen Bradley, Judith Yargawa, Veronica Pingray, Gabriela Cormick, Adrienne Gordon, Vicki Flenady, Erzsébet Horváth‐Puhó, Henrik Toft Sørensen, Luule Sakkeus, Liili Abuladze, Mohammed Heidarzadeh, Narjes Khalili, Khalid A. Yunis, Ayah Al Bizri, Shamala D. Karalasingam, Ravichandran Jeganathan, Arturo Barranco, Aimée E. van Dijk, Lisa Broeders, Fawzya Alyafei, Mai AlQubaisi, Neda Razaz, Jonas Söderling, Lucy K. Smith, Ruth J. Matthews, Rachael Wood, Kirsten Monteath, Isabel Pereyra, Gabriella Pravia, Sarka Lisonkova, Qi Wen, Joy E. Lawn, Hannah Blencowe, the National Vulnerable Newborn Collaborative Group for Stillbirths, Veronica Pingray, Gabriela Gabriela, José Belizan, Luz Gibbons, Carlos Guevel, Vicki Flenady, Adrienne Gordon, Kara Warrilow, Harriet Lawford, Erzsébet Horváth‐Puhó, Henrik T. Sørensen, Luule Sakkeus, Liili Abuladze, Khalid A. Yunis, Ayah Al Bizri, Pascale Nakad, Shamala Karalasingam, J. Ravichandran, R. Jeganathan, Nurakman Binti Baharum, Lorena Suárez‐Idueta, Arturo Barranco Flores, Jesus Felipe Gonzalez Roldan, Sonia Lopez Alvarez, Lisa Broeders, Aimée E. van Dijk, Fawzia Alyafei, Mai AlQubaisi, Tawa O. Olukade, Hamdy A. Ali, Mohamad Rami Alturk, Neda Razaz, Jonas Söderling, Lucy K. Smith, Bradley N. Manktelow, Ruth J. Matthews, Elizabeth Draper, Alan Fenton, Jennifer J. Kurinczuk, Rachael Wood, Celina Davis, Kirsten Monteath, Samantha Clarke, Isabel Pereyra, Gabriella Pravia, Sarka Lisonkova, Qi Wen, Joy E. Lawn, Hannah Blencowe, Eric Ohuma, Yemi Okwaraji, Judith Yargawa, Ellen Bradley, Robert Black, Joanne Katz, Dan Erchick, Elizabeth Hazel, Mike Diaz, Anne C. C. Lee

**Affiliations:** ^1^ Maternal, Adolescent, Reproductive & Child Health (MARCH) Centre London School of Hygiene & Tropical Medicine London UK; ^2^ Mexican Society of Public Health Mexico City Mexico; ^3^ Institute for Clinical Effectiveness and Health Policy Ciudad Autónoma de Buenos Aires Argentina; ^4^ Centro de Investigaciones en Epidemiología y Salud Pública, National Scientific and Technical Research Council (CONICET) Ciudad Autónoma de Buenos Aires Argentina; ^5^ Faculty of Medicine and Health University of Sydney Sydney New South Wales Australia; ^6^ Department of Clinical Epidemiology Aarhus University and Aarhus University Hospital Aarhus N Denmark; ^7^ School of Governance, Law and Society, Estonian Institute for Population Studies Tallinn University Tallinn Estonia; ^8^ Population Research Unit Väestöliitto Finland; ^9^ Paediatrics Department Alzahra Hospital Iran Tabriz Iran; ^10^ Department of Community and Family Medicine, Preventive Medicine and Public Health Research Centre, Psychosocial Health Research Institute, School of Medicine Iran University of Medical Sciences Tehran Iran; ^11^ Division of Neonatology, Department of Pediatrics and Adolescent Medicine American University of Beirut Beirut Lebanon; ^12^ Department of Obstetrics and Gynaecology, Faculty of Medicine University of Cyberjaya Cyberjaya Malaysia; ^13^ Department of Obstetrics & Gynaecology Malaysia Monash Medical School Johor Bahru Malaysia; ^14^ Directorate of Health Information Ministry of Health Mexico City Mexico; ^15^ Perined Utrecht The Netherlands; ^16^ Department of Paediatrics Hamad General Hospital Doha Qatar; ^17^ NICU, Women Wellness and Research Centre Doha Qatar; ^18^ Clinical Epidemiology Division, Department of Medicine Solna Karolinska Institute Stockholm Sweden; ^19^ Department of Population Health Sciences, College of Life Sciences University of Leicester Leicester UK; ^20^ Public Health Scotland Edinburgh UK; ^21^ Usher Institute University of Edinburgh Edinburgh UK; ^22^ Pregnancy, Birth and Child Health Team Public Health Scotland Edinburgh UK; ^23^ Faculty of Health Sciences Catholic University of Maule Curicó Chile; ^24^ Department of Wellness and Health Catholic University of Uruguay Montevideo Uruguay; ^25^ Department of Obstetrics & Gynaecology University of British Columbia Vancouver British Columbia Canada; ^26^ School of Population and Public Health University of British Columbia Vancouver British Columbia Canada

**Keywords:** gestational age, newborn, pregnancy, premature birth, preterm, stillbirths

## Abstract

**Objective:**

To examine the contribution of preterm birth and size‐for‐gestational age in stillbirths using six ‘newborn types’.

**Design:**

Population‐based multi‐country analyses.

**Setting:**

Births collected through routine data systems in 13 countries.

**Sample:**

125 419 255 total births from 22^+0^ to 44^+6^ weeks’ gestation identified from 2000 to 2020.

**Methods:**

We included 635 107 stillbirths from 22^+0^ weeks’ gestation from 13 countries. We classified all births, including stillbirths, into six ‘newborn types’ based on gestational age information (preterm, PT, <37^+0^ weeks versus term, T, ≥37^+0^ weeks) and size‐for‐gestational age defined as small (SGA, <10th centile), appropriate (AGA, 10th–90th centiles) or large (LGA, >90th centile) for gestational age, according to the international newborn size for gestational age and sex INTERGROWTH‐21st standards.

**Main outcome measures:**

Distribution of stillbirths, stillbirth rates and rate ratios according to six newborn types.

**Results:**

635 107 (0.5%) of the 125 419 255 total births resulted in stillbirth after 22^+0^ weeks. Most stillbirths (74.3%) were preterm. Around 21.2% were SGA types (PT + SGA [16.2%], PT + AGA [48.3%], T + SGA [5.0%]) and 14.1% were LGA types (PT + LGA [9.9%], T + LGA [4.2%]). The median rate ratio (RR) for stillbirth was highest in PT + SGA babies (RR 81.1, interquartile range [IQR], 68.8–118.8) followed by PT + AGA (RR 25.0, IQR, 20.0–34.3), PT + LGA (RR 25.9, IQR, 13.8–28.7) and T + SGA (RR 5.6, IQR, 5.1–6.0) compared with T + AGA. Stillbirth rate ratios were similar for T + LGA versus T + AGA (RR 0.7, IQR, 0.7–1.1). At the population level, 25% of stillbirths were attributable to small‐for‐gestational‐age.

**Conclusions:**

In these high‐quality data from high/middle income countries, almost three‐quarters of stillbirths were born preterm and a fifth small‐for‐gestational age, with the highest stillbirth rates associated with the coexistence of preterm and SGA. Further analyses are needed to better understand patterns of gestation‐specific risk in these populations, as well as patterns in lower‐income contexts, especially those with higher rates of intrapartum stillbirth and SGA.

## 1 INTRODUCTION

The World Health Organization (WHO) defines stillbirth as the loss of a baby during pregnancy at or after 22^+0^ weeks of gestation or, if gestational age is not available, weighing 500 g or more (Table 1).^1^ Global estimates are only available for late gestation stillbirths. These estimate that 1.9 million babies were stillborn after 28^+0^ weeks’ gestation in 2021.^2^ Stillbirth is associated with large emotional toll on affected women, families, health workers and society, representing a substantial loss of human capital.^3^ Importantly, most of these deaths are preventable through improved access to high‐quality antenatal and intrapartum care.^4^, ^5^

The Every Newborn Action Plan set a target of 12 or fewer late gestation stillbirths per 1000 total births by 2030.^6^, ^7^ According to the latest estimates, if current trends persist, 56 countries will not meet this stillbirth rate target.^2^, ^8^ The countries needing most acceleration to meet these targets are in sub‐Saharan Africa and South Asia, where stillbirth rates are highest, but data availability lowest. Further epidemiological data are needed to understand drivers of stillbirth to inform investments for programmatic action towards ending these frequently preventable deaths.^6^ Data on stillbirths are now available from 173 countries (with data from 138 countries meeting quality inclusion criteria for UN estimates). Many middle‐ and higher‐income countries have individual‐level data records that can enable more detailed assessments, which could lead to insights in patterns of stillbirth to inform interventions.

Stillborn babies are more likely to be growth‐restricted (assessed at birth using the proxy of small for gestational age [SGA, <10th centile]) or preterm (<37^+0^ weeks’ gestational age) and therefore more likely to be low birthweight (LBW, <2500 g) than are live‐born peers.^9^, ^10^ Previous studies have shown that babies compromised through poor fetal growth are at higher risk of stillbirth – both prior to the start of labour (antepartum stillbirth) and during labour (intrapartum stillbirth).^11^, ^12^

LBW has traditionally been used as the main marker of vulnerability. Recent work recognising the two underlying pathways to LBW – short gestation and fetal growth restriction – has proposed the concept of vulnerable ‘newborn types’, with an initial focus primarily on live births.^13^, ^14^ No studies to date have sought to categorise stillbirths using these types.

Ashorn et al. called for a better description of the prevalence and mortality risk of ‘newborn types’ based on length of gestation and size for gestational age at birth to delineate vulnerability.^13^ These ‘newborn types’ could also assist in the identification of babies at the highest risk of complications, to help better understand biological mechanisms, to inform more targeted and innovative interventions, and to accelerate progress towards global LBW and neonatal mortality reduction targets. Accompanying papers in this supplement have described the prevalence and mortality risk by ‘newborn type’ among live births.^15^, ^16^ These have demonstrated the association between newborn type and neonatal mortality risk with the greatest risk for preterm ‘newborn types’, especially with co‐existence of preterm and SGA.

This paper aims to assess the use of this classification to provide a more granular description of stillbirths. In this study, we examined the distribution of stillbirths by these ‘newborn types’.

**TABLE 1:** Key findings.

**1. What was known?** Stillbirth (pregnancy loss after 22^+0^ weeks) is a devastating outcome. Global estimates indicating 1.9 million late gestation stillbirths (≥28^+0^ weeks) worldwide in 2021 underestimate the overall burden because the estimate does not include early gestation stillbirths. Many of the pathways to stillbirth result in fetal death before term (preterm stillbirth, <37^+0^ weeks of gestational age). In addition, babies with fetal growth restriction (frequently assessed using the proxy small for gestational age (SGA, <10th centile)) are at higher risk of stillbirth than their appropriately grown peers. Stillbirths are therefore more likely to be low birthweight (LBW, <2500 g). Being large for gestational age (LGA, >90th centile) at term may also be associated with increased risk of stillbirth.
**2. What was done that is new?** Combining information on gestational age (preterm [PT], or term [T]) and attained size for‐gestational‐age (small‐for‐gestational‐age [SGA], appropriate‐for‐gestational age [AGA], large‐for‐gestational age [LGA]) we defined six ‘newborn types’: four small (PT + SGA, PT + AGA, PT + LGA, T + SGA), one large (T + LGA), and one reference (T + AGA). We compiled livebirth and stillbirth data from 15 high‐ and middle‐income countries as part of the Vulnerable Newborn Collaboration. A total of 124,784,148 livebirths and 635,107 stillbirths ≥22^+0^ weeks from 13 countries between 2000 and 2020 met the inclusion criteria. We examined the distribution of stillbirths by these ‘newborn types’, and calculated type‐specific stillbirth rates and rate ratios.
**3. What was found?** Most stillbirths (74.3%) were preterm, compared to fewer than 1‐in‐10 (8.9%) livebirths. A fifth (21.2%) of stillbirths were SGA compared to 1‐in‐20 (5.3%) livebirths. Preterm SGA had 81.1 times higher stillbirth rates compared to term AGA (Rate ratio [RR] = 81.1, interquartile range [IQR], 68.8, 118.8). Overall, preterm types had a 25.3 times higher stillbirth rate than term types (RR = 25.3, IQR, 20.3, 31.2). At the population level, over a quarter of stillbirths (25%) were attributable to being SGA, indicating a substantial impact of growth restriction on stillbirth in these settings. 14.0% of stillbirths and 17.7% of livebirths were LGA. There was no evidence of increased stillbirth rates for LGA types. The distribution of these ‘newborn types’ are similar amongst stillbirths and neonatal deaths.
**4. What next?** Categorisation of all births, including stillbirths, into these ‘newborn types’ was analytically possible using routinely collected data in these 13 upper‐middle‐ or high‐income contexts and led to programmatic relevant findings. However, as the majority (98%) of the world's stillbirths are in low‐and middle‐income countries, more data are needed to improve understanding of patterns in stillbirths in a wider range of contexts, especially in settings with higher rates of intrapartum stillbirth and those with very high SGA rates such as South Asia. Further analyses, including assessing gestational age‐specific risk, could provide more information on pathways to stillbirth and enable targeted interventions to underlying causes such as infection and obstetric complications. When analysing these vulnerability pathways, omitting stillbirths neglects an important part of the burden and its effects on families and society.

## 2 METHODS

### 2.1 Data source

A detailed description of how data were collated has been published in detail elsewhere.^14^, ^16^ In brief, 15 of the 23 countries participating in the Vulnerable Newborn Measurement collaboration provided information on stillbirths and were considered in these analyses. Data from the 15 countries were compiled for all births (live births and stillbirths) from 2000 to 2020, including more than 138 country‐years. Each country team analysed their datasets with standardised codes in statistical programs STATA, R or SAS using programming developed centrally by the London School of Hygiene & Tropical Medicine (LSHTM), with summary tables shared online through a secured data hub. In accordance with the International Classification of Diseases, stillbirths were defined as fetal deaths at ≥22^+0^ weeks of gestation (Table S1a).^1^ Sensitivity analyses were undertaken to include only late gestation stillbirths at ≥28^+0^ weeks’ gestation.

**FIGURE 1:**
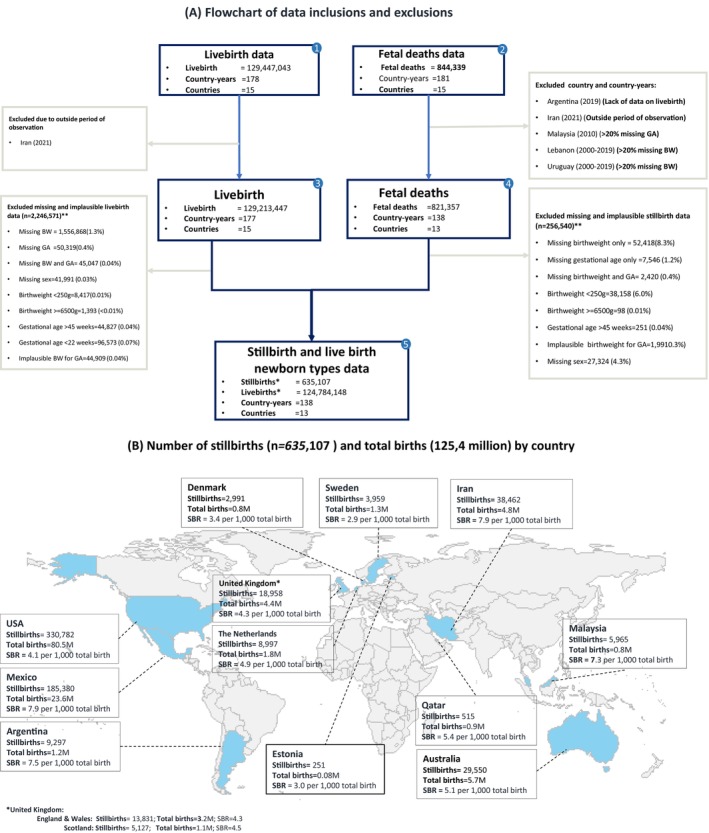
Input data for stillbirth analyses, 2000–2020. (A) Flowchart of data inclusions and exclusions. *For the sensitivity analysis: 232 488 stillbirths and 612 436 live births at 22–27 weeks’ gestational age were excluded. Total number of births at ≥28 weeks: 402 619 late gestation stillbirths, 124 171 712 live births. **Due to overlaps of missing and implausible data, the total excluded values do not add up to the difference between box 3 and box 4 and between box 3 and box 5. (B) Number of stillbirths (635 107) and total births (125.4 million) by country. *UK: England & Wales: stillbirths = 13 831, total births = 3.2 million, SBR = 4.3; Scotland: stillbirths = 5127, total births = 1.1 million, SBR = 4.5. Map legend shows the distribution of 125.4 million total births (124.7 million live births and 635 107 stillbirths at ≥22^+0^ weeks) with information to classify by ‘newborn types’ included in this study.

Individual birth records missing birthweight, gestational age and/or sex were excluded as it was not possible to assess size‐for‐gestational age (Figure 1A). Birth records with gestational age <22^+0^ or >44^+6^ weeks or implausible combinations of birthweight and gestational age (defined as birthweight ±5 standard deviations from the mean birthweight for gestational age) were also excluded.

Data quality assessments were performed by estimating the level of missingness of core variables and of implausible values by each country‐year (Table S1b). We evaluated the plausibility of the stillbirth dataset by comparing the absolute differences between the calculated late gestation stillbirth rate (SBR; ≥28^+0^ weeks) in our data and the nationally reported SBR for late gestation stillbirth rates (Table S1c).^8^ We excluded country‐years with >20% missing birthweight or gestational age data.

Findings are reported in accordance with the Reporting guidelines of studies Conducted using Observational Routinely collected Data, the RECORD statement (Table S2). Ethics approval for all participants is presented in Table S3.

### 2.2 Construction of ‘newborn types’ as exposure indicators

Consistent with the approach previously taken for live births,^15^, ^17^ each birth was categorised into six mutually exclusive ‘newborn types’. First, we categorised every birth record as preterm (<37^+0^ weeks [PT]) or term (≥37^+0^ weeks [T]). Next, we classified births by size‐for‐gestational age defined as small (SGA, <10th centile), appropriate (AGA, 10th–90th centiles), or large (LGA, >90th centile) for gestational age using a modified version of the INTERGROWTH‐21st international newborn size for gestational age and sex standards extended to include all births from 22^+0^ to 44^+6^ weeks.^18^ We created a set of a six ‘newborn types’ based on the combination of PT or T and size‐for‐gestational age: four small (PT + SGA, PT + AGA, PT + LGA, T + SGA), one large (T + LGA), and one reference (T + AGA).

### 2.3 Data statistical analysis

Among the included records, measures were calculated and summarised with the median and IQR.

#### 2.3.1 Distribution of stillbirths by type

The number of stillbirths reported for each type was divided by the total number of stillbirths per 100. This calculation was repeated for live births and neonatal deaths (death during the first 28 days of life following a live birth) and the distributions compared.

#### 2.3.2 Type‐specific stillbirth rate

Stillbirth rates for each type were calculated as the number of stillbirths in the group divided by the total number of births in that group expressed as stillbirths per 1000 total births (e.g. number of stillbirths between PT + SGA divided by number of total births between PT + SGA per 1000).

#### 2.3.3 Stillbirth type‐specific rate ratio

Rate ratios were calculated as the stillbirth rate in each type group, divided by the stillbirth rate in the reference group (T + AGA). These were calculated for each ‘newborn type’ and also for preterm types combined.

#### 2.3.4 Population attributable fraction (PAF)

The prevalence of SGA type was multiplied by the rate ratio in each type divided by the sum of the prevalence of SGA types multiplied by the rate ratio of all ‘newborn types’ in the population. We calculated PAF only for SGA types, as a proxy for fetal growth restriction, as fetal growth restriction is a potential pathway to stillbirth.

### 2.4 Sensitivity analysis

In view of the WHO recommendation for the use of late gestation stillbirth (≥28^+0^ weeks) for international comparisons and the potential large variations in ascertainment capture and reporting of early gestation stillbirth (22^+0^–27^+6^ weeks), we carried out a sensitivity analysis to explore whether the distribution of stillbirth and stillbirth rate ratios differed if only late gestation stillbirths (28^+0^–44^+0^ weeks) were included.

## 3 RESULTS

### 3.1 Data quality assessment

Data were assessed from 15 national datasets collected between 2000 and 2020. We excluded country‐years with ≥20% missing birthweight or gestational age (Lebanon in 2000–2019, Uruguay in 2000–2019 and Malaysia in 2010); missing information on live births (Argentina in 2019) and those which lay outside the study period (Iran in 2021) (Figure 1A). Overall, 15.4% (27/175) and 8.0% (14/175) of country‐years had ≥20% missing birthweight data and missing gestational age, respectively, and were excluded (Table S1b).

Data from 13 countries representing 125 419 255 total births (124 784 148 live births and 635 107 stillbirths) were included. Of the stillbirths, 232 488 were early gestation (22^+0^–27^+6^ weeks) and 402 619 late gestation (≥28^+0^ weeks). Data from a wide geographical range of high‐income and middle‐income countries were included (Figure 1B).

The overall stillbirth rate was 5.0 per 1000 total births, with the highest rates in Iran, Mexico and Argentina (7.9, 7.8 and 7.4 per 1000 total births, respectively). The lowest stillbirth rate was observed in Sweden, with 2.9 per 1000 total births.

### 3.2 Distribution of stillbirths by newborn type

**FIGURE 2:**
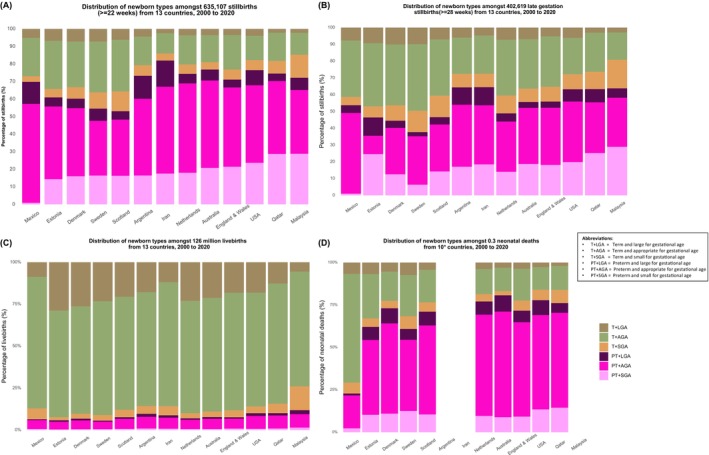
Distribution of ‘newborn types’ among: (A) all stillbirths (see Table S4a for country‐level data), (B) late gestation stillbirths (see Table S4b for country‐level data), (C) live births, (D) neonatal deaths, 2000–2020. *Data not available from Argentina, Iran or Malaysia.

The distribution of stillbirths according to the six ‘newborn types’ showed that most stillbirths (74.4%) were preterm types (PT + SGA [16.2%], PT + AGA [48.3%], PT + LGA [9.9%]) (Table 2; Figure 2A). Less than a fifth of stillbirths were T + AGA (16.7%), with around one in 20 T + SGA and T + LGA (5.0% and 4.2%, respectively) (Table 2).

**TABLE 2:** Stillbirth rate and rate ratio by newborn type for all stillbirths (≥22^+0^ weeks), 2000–2020.

Measurements	Newborn types					
	PT + SGA	PT + AGA	PT + LGA	T + SGA	T + AGA	T + LGA
Total births, *n* (%)	982 390 (0.8)	9 013 016 (7.2)	1 677 042 (1.3)	5 743 330 (4.6)	87 536 596 (69.8)	20 466 881 (16.3)
Stillbirths, *n* (%)	102 831 (16.2)	305 995 (48.3)	62 663 (9.9)	31 557 (5.0)	105 532 (16.7)	26 529 (4.2)
Stillbirth distribution, %, median (IQR)	19.7 (16.2–23.6)	44.6 (37.7–49.1)	7.0 (5.4–8.7)	5.8 (4.2–10.4)	18.2 (14.1–27.6)	4.0 (3.5–5.8)
Stillbirth rate per 1000 total births, median (IQR)	116.2 (91.6–130.9)	30.5 (22.9–40.5)	28.2 (21.2–36.1)	6.8 (5.6–9.0)	1.3 (1.1–1.8)	1.0 (0.8–1.5)
Stillbirth rate ratio, median (IQR)	81.1 (68.8–118.8)	25.0 (20.0–34.3)	25.9 (13.8–28.7)	5.6 (5.1–6.0)	1 (Reference)	0.7 (0.7–1.1)

There was substantial country‐level variation in the distribution of ‘newborn types’ among stillbirths. Overall, among all stillbirths ≥22^+0^ weeks, the median PT + SGA was 19.7% (IQR 16.2–23.6) (ranging from 0.9% in Mexico to 28.8% in Malaysia); median PT + AGA 44.6% (IQR 37.7–49.1) (ranging from 31.1% in Sweden to 56.4% in Mexico); median PT + LGA 7.0% (IQR 5.4–8.7) (ranging from 4.4% in Qatar and England & Wales to 14.8% in Iran); median T + SGA 5.8% (IQR 4.2–10.4) (ranging from 3.2% in Mexico to 13.1% in Malaysia); median T + AGA 18.2% (IQR 14.1–27.6) (ranging from 11.6% in Iran to 92.5% in Scotland); median T + LGA 4.0% (IQR 3.5–5.8) (ranging from 2.0% in Qatar to 7.2% in Denmark) (Table 2; Table S4a). Almost half of all stillbirths were preterm and AGA, with the highest percentages in Mexico 56.4% followed by the Netherlands (50.9), Australia (49.5%), Iran (49.5%), England & Wales (45.3%) and USA (44.2%). Malaysia reported the highest prevalence of preterm and SGA stillbirth (28.8%), followed by Qatar (28.0%) and USA (23.7%). In contrast, Denmark, Sweden and Scotland reported relatively high percentages of term and LGA stillbirth – 7.2%, 7.1% and 5.9%, respectively (Figure 2A; Table S4a).

#### 3.2.1 Comparison of distribution of ‘newborn types’ for live births and neonatal deaths

A similar pattern to the distribution of ‘newborn types’ for stillbirths was observed for neonatal deaths. Around 75% of neonatal deaths in all countries, apart from Mexico, were preterm (Figure 2C). In contrast, most live births (90%) were born at term (T + AGA [69.8%], T + LGA [16.4%], T + SGA [4.6%]), with the remaining 10% preterm (PT + SGA [0.8%], PT + AGA [7.2%], PT + LGA [1.3%]; Figure 2D).

### 3.3 Rates of stillbirth by type

The overall stillbirth rate (including all stillbirths ≥22^+0^ weeks) for the study period was 5.0 per 1000 total births. Stillbirth rates were highest for preterm ‘newborn types’: PT + SGA (median 116.2 stillbirths per 1000 total births, IQR 91.6–130.9), PT + AGA (median 30.5, IQR 22.9–40.5), and PT + LGA (median 28.2, IQR 21.2–36.1), followed by T + SGA (median 6.8, IQR 5.6–9.0), T + AGA (median 1.3, IQR 1.1–1.8) and T + LGA (median 1.0, IQR 0.8–1.5) (Table 2). At country‐level, the highest stillbirth rates among the PT + SGA types were observed in Australia (SBR 154.1, 95% CI 153.7–154.4) followed by Iran (SBR 149.2, 95% CI 149.0–149.4) and Qatar (SBR 132.2, 95% CI 131.4–133.2) (Table S4a). Mexico, Iran and Argentina had the highest three stillbirth rates among the PT + AGA types (SBR 77.3; 95% CI 77.2–77.4; SBR 57.2, 95% CI 57.0–57.5; and SBR 44.9, 95% CI 44.9–44.9, respectively; Table S4a).

### 3.4 Stillbirth rate ratios by ‘newborn type’

**FIGURE 3:**
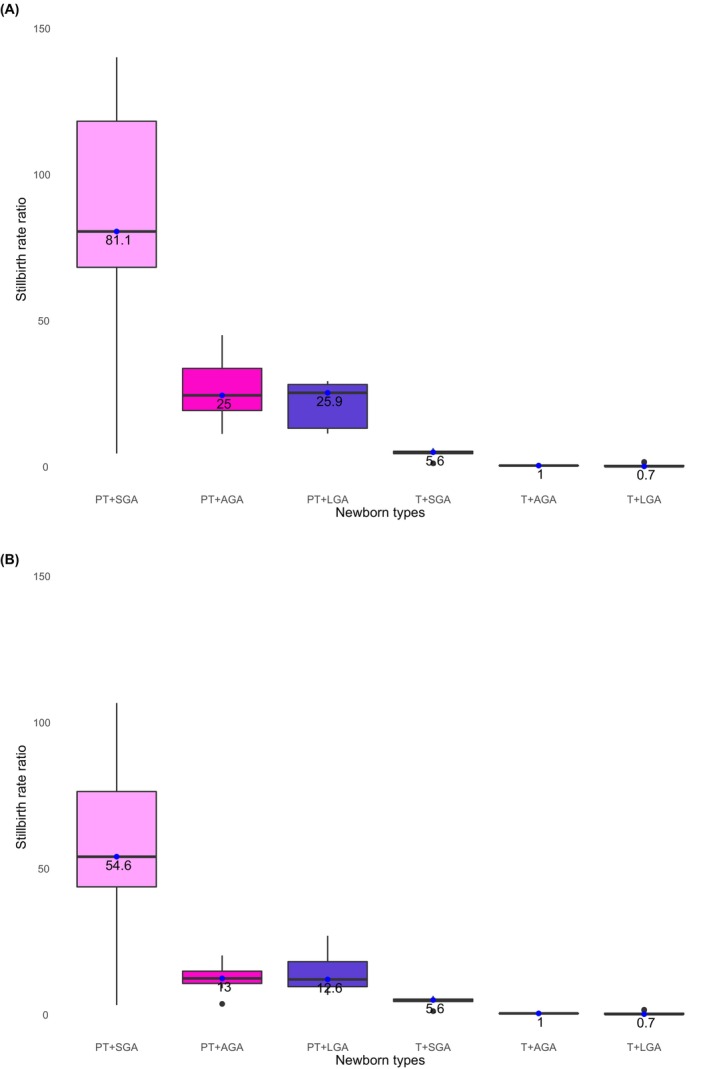
(A) Stillbirth rate ratio by ‘newborn types’ among all stillbirths (≥22^+0^ weeks), 2000–2020. (B) Stillbirth rate ratio by ‘newborn types’ among late gestation stillbirth (≥28^+0^ weeks), 2000–2020. Each point represents the stillbirth rate ratio, box plots summarise median values and IQR (25th and 75th percentiles) (A: countries = 13, *n* = 635 107; see Table S4a for country‐level data. B: countries = 13, *n* = 402 619; see Table S4b for country level data).

Compared with T + AGA, the median stillbirth rate ratio was more than 80‐fold higher (median RR 81.1, IQR 68.8–118.8) for babies with the coexistence of preterm and SGA, over 20‐fold higher for those PT + LGA (median RR 25.9, IQR 13.8–28.7) or PT + AGA (median RR 25.0, IQR 20.0– 34.3), and five‐fold higher for babies T + SGA (median RR 5.6, IQR 5.1–6.0) (Table 2; Figure 3A).

At country‐level, the highest stillbirth rate ratio for PT + SGA was observed in USA (RR 140.9, 95% CI 140.8–141.0), followed by Australia (RR 133.2, 95% CI 133.0–133.5) and Qatar (RR 117.4.8, 95% CI 116.6–118.1) (Table S4a). Iran, Netherlands and Mexico had the highest stillbirth rate ratios for PT + AGA types (RR 45.4, 95% CI 45.3–45.5; RR 22.6, 95% CI21.9–

23.2; RR 35.3, 95% CI 35.1, 35.5, respectively; Table S4a).

### 3.5 Contribution of SGA to stillbirths (PAF)

At the population level, a quarter (25%) of stillbirths were attributable to being SGA before term (PT + SGA median PAF 20.0, IQR 16.0–24.0), with an additional 5% attributable to SGA at term (T + SGA median PAF 5, IQR 4–8).

### 3.6 Sensitivity analyses

Preterm types remained the dominant type even when only late gestation stillbirths were included, with around half of all stillbirths being preterm (Table 3; Figure 2B).

**TABLE 3:** Stillbirth rate and rate ratio by newborn type for all stillbirths (≥28^+0^ weeks), 2000–2020.

Measurements	Newborn types					
	PT + SGA	PT + AGA	PT + LGA	T + SGA	T + AGA	T + LGA
Total births, *n* (%)	878 893 (0.7)	8 385 553 (6.7)	1 563 078 (1.3)	5 743 330 (4.6)	87 536 596 (70.3)	20 466 881 (16.4)
Stillbirths, *n* (%)	55 671 (13.7)	157 728 (39.0)	25 602 (6.3)	31 557 (7.8)	105 532 (26.0)	26 529 (6.5)
Stillbirth distribution, %, median (IQR)	15.9 (11.7–19.4)	31.4 (28.1–35.9)	4.5 (3.5–6.9)	9.2 (7.4–12.4)	29.4 (22.1–34.7)	6.3 (5.4–7.9)
Stillbirth rate per 1000 total births, median (IQR)	69.78 (54.1–89.0)	15.4 (13.5–19.9)	14.6 (10.7–23.5)	6.8 (5.6–9.0)	1.3 (1.1–1.8)	1.0 (0.8–1.5)
Relative risk, median (IQR)	54.6 (44.3–77.0)	13.0 (11.3–15.4)	12.6 (10.2–18.7)	5.6 (5.1–5.9)	1 (Reference)	0.7 (0.7–1.1)

A similar pattern in stillbirth rate and rate ratios was observed when only late gestation stillbirths were included, although the late gestation preterm ‘newborn type’‐specific stillbirth rates were around two‐thirds of those for all births from 22^+0^ weeks and the stillbirth rate ratios for the PT + AGA and PT + LGA and half of those for all births from 22^+0^ weeks (Table 3).

## 4 DISCUSSION

### 4.1 Main findings

This paper, including analyses of more than half a million babies from 13 countries stillborn between 2000 and 2020, has provided the first multi‐country description of stillbirths using this novel classification by ‘newborn type’ combining attained size‐for‐gestational age and preterm or term. This classification goes beyond the traditional cut‐offs and enables assessment of the contribution of preterm, SGA and their combination. This has been shown to be useful for live births in identifying risk of neonatal death.^15^, ^19^ Our results showed the overlap between preterm birth and stillbirth, with around three‐quarters of all stillbirths in these settings born preterm, compared with just 9.2% of live births. A fifth (21.2%) of stillbirths were SGA at birth, a substantially higher proportion than for live births (5.5%). The stillbirth rate ratios were highest for the combination of preterm and SGA compared with T + AGA. No additional stillbirth risk was found for term LGA babies compared with term AGA babies.

### 4.2 Interpretation

Stillbirths are strongly associated with gestational ages <37^+0^ weeks.^20^ In this study, around 75% of stillbirths were preterm, slightly higher than that reported in a recent study in six low‐ and middle‐income countries, which reported 60% of stillbirths preterm.^21^

We found the largest difference in stillbirth rates compared with T + AGA in all countries was for births that were both preterm and SGA (as a proxy for being growth‐restricted), followed by those PT + AGA, or PT + LGA. The increased risk for PT + LGA compared with appropriately grown term births is likely to be driven by low gestational age rather than large size for gestational age. Overall stillbirth rates for preterm types were 25 times higher than for term types. Consistent with previous research,^22^ the present study found that those SGA at term were more likely to be stillborn than their appropriately grown peers.

At population level, SGA (diagnosed at birth) contributed to around 25% of all stillbirths in these 13 countries, with relatively high levels of pregnancy monitoring and interventional obstetrics, including provider‐initiated delivery following in utero diagnosis of severe fetal growth restriction. This is higher than the 11% population attributable risk reported in a previous study of eight high and middle‐income countries. However, that study included only antepartum stillbirths from low‐risk women and may not be generalisable to the whole population.^23^

Understanding the population‐level scale of the impact of fetal growth on stillbirth is crucial, as stillbirths associated with fetal growth restriction are preventable with improved antenatal screening.^24^ However, there is a balance of risks between detecting fetal growth restriction and acting to prevent stillbirth, versus increasing preterm birth and associated complications.^25^ This balance of risks is even more pertinent in low‐resource settings where full neonatal intensive care is less likely to be available. A recent multi‐country study (Ghana, India, Kenya, Rwanda and South Africa) found routine Doppler screening in a low‐risk obstetric population an effective tool for reducing stillbirth rates.^26^ In France, antenatal detection of fetal growth restriction (FGR) was found to be protective against stillbirth, but despite detection of FGR, over 40% of stillbirths occurred in SGA babies.^27^

There is a major focus on small size at birth; however, increasing evidence indicates that large for gestational age, which may be associated with the maternal metabolic environment, is also associated with an increased risk of stillbirth.^28^, ^29^ In this study we found no increased risk of stillbirth in term babies who were LGA at birth compared with AGA, although this may be partly because the included populations had very low rates of post‐term delivery, where the risks associated with LGA may be greater. This finding differs from that of previous studies where the risk of stillbirth after 36^+0^ weeks’ gestation was higher for LGA than for AGA pregnancies.^22^, ^28^, ^30^ However the use of the INTERGROWTH‐21st newborn standard may also account for these differences, as it is known to left‐shift the centile distribution compared with national charts used in other studies.^31^

### 4.3 Strength and limitations

A strength of our analyses is the large sample size combining data from across 13 countries, with high data completeness and other measure of data quality. This has enabled exploration of associations with gestational age, by attained size for‐gestational‐age, and across time.

There are also limitations. Importantly, this study uses size‐for‐gestational age at birth as a proxy for fetal growth restriction (FGR). FGR is defined as the failure of the fetus to meet its growth potential due to a pathological factor, most commonly placental dysfunction.^24^ FGR is diagnosed by a drop of estimated fetal weight (EFW) centile on serial ultrasound measurement. In practice, this is not always available, and clinicians may rely on single ‘snapshot’ EFW assessment to define whether a baby is SGA in utero – and are hence not able to differentiate whether an SGA baby is small due to predetermined growth potential or growth‐faltering. In this study, the use of size‐for‐gestational age at birth rather than EFW in utero may, in the rare cases where there is a prolonged period between fetal death and delivery, result in babies appropriately grown until the time of fetal death being classified at birth as SGA. Globally, nearly half of all stillbirths occur intrapartum.^8^ However, in some cases of antepartum stillbirths “a prolonged period between fetal death and delivery [may] result in babies appropriately grown until the time of fetal death being classified at birth as SGA”. This may be particularly relevant in high burden settings where intensive obstetric monitoring is less available. In addition, the reduction in birthweight due to postmortem desiccations may further exacerbate the association between SGA and stillbirth.^32^

Secondly, to seek to provide comparability with live births, these ‘newborn types’ were based on the comparison with T + AGA. However, using a single dichotomous preterm versus term categorisation for stillbirths may not provide the level of granularity required and, importantly, using such an approach it was not possible to estimate gestation‐specific risk using a fetuses‐at‐risk approach.^33^

The comparability of results may be affected by the variation in gestational age assessment methods used (last menstrual period, different best obstetric estimates, ultrasound and the timing of ultrasound assessment). In addition our findings may also be affected by variations in stillbirth definition used by countries and whether elective terminations of pregnancy are combined with stillbirths for reporting purposes (Table S5).^34^ It is well recognised that the reporting of births and misclassification between stillbirth and very early neonatal death is more common around the clinician's perceptions of limits of viability and the thresholds of reporting in any given setting^4^, ^35^, ^36^ Therefore shifting the threshold of reporting down to require reporting of all fetal deaths from 20^+0^ weeks will improve capture of all stillbirths from 22^+0^ weeks as defined by WHO.^1^ However, most countries only routinely recorded stillbirths from 22^+0^ weeks in their data system, with some only reporting from 24^+0^ weeks (Table S5). In the latter cases, although data were provided for this study on stillbirth at 22 or 23 weeks, there may be under‐capture, as reporting of these deaths is not mandatory. Hence, we undertook a sensitivity analysis including only late gestation stillbirths at ≥28^+0^ weeks (63.4% of all stillbirths). This showed a similar pattern to the main analyses, with the highest rates and rate ratios for the preterm types and, as expected, the stillbirth rate and rate ratios by ‘newborn types’ were lower for all groups when considering only late gestation stillbirths compared with all stillbirths at ≥22^+0^ weeks.

Furthermore, despite around 98% of global stillbirths occurring in low‐ and middle‐income countries, high‐quality routine individual‐level data on stillbirths from these countries are lacking and it was not possible to include these countries in this analysis.

Further research is required to assess the use of these ‘newborn types’ for stillbirths in higher burden contexts, especially those with high rates of SGA, notably South Asia.^14^ In addition, assessing risk by more detailed gestational age categories using a fetuses‐at‐risk approach, including data on labour‐type (spontaneous versus provider‐initiated), and combining these analyses with analyses of neonatal deaths could enable improved understanding of the epidemiology and provide data to target interventions, especially in settings with high levels of pregnancy monitoring and interventional obstetrics.^37^

## 5 CONCLUSION

Our study provides the first multi‐country analysis of ‘newborn types’ for stillbirths. Where individual level data are available categorisation of all births, including stillbirths, into these ‘newborn types’ was analytically possible using routinely collected data in these 13 upper‐middle‐ or high‐income contexts and led to programmatic relevant findings.

Preterm stillbirth accounted for more than three‐quarters of all stillbirths in these high‐quality data from high/middle income countries. SGA is also associated with stillbirth, especially in combination with being preterm. More analyses of these ‘newborn types’ across a range of mortality contexts, and extending gestation and size risk assessment using a fetuses‐at‐risk approach could provide more information on pathways to stillbirth and enable better targeting of interventions to underlying causes such as infections and obstetric complications.

## AUTHOR CONTRIBUTIONS

The Vulnerable Newborn Measurement Collaboration was conceptualised by Joy Lawn and Bob Black. All collaborators contributed to the design of the study protocol. YBO, HB, EOO and JEL developed the detailed research questions and overall analysis plan for this paper. These were refined with inputs from the wider Vulnerable Newborn Measurement Collaboration. Analysis was undertaken by YBO, LS‐I, HB and EOO provided statistical oversight. The paper was drafted by YBO, LS‐I and HB with EOO and JEL. All authors reviewed and agreed on the final version for publication.

## CONFLICT OF INTEREST STATEMENT

None declared.

## FUNDING INFORMATION

The Children's Investment Fund Foundation, prime grant 1803‐02535. The funders had no role in the study design, data collection, analysis, interpretation of the data, or the decision to submit for publication.

## ETHICS STATEMENT

The Vulnerable Newborn Measurement Collaboration was granted ethical approval from the Institutional Review Boards of the London School of Hygiene & Tropical Medicine (ref: 22858) and Johns Hopkins University. All the 13 country teams had ethical approval for use of data or exemptions based on current remit (Table S5).

## Supporting information


**DAppendixata S1:** Supporting information

## Data Availability

Data sharing and transfer agreements were jointly developed and signed by all collaborating partners. The pooled summary table data generated during the current study have been deposited online with data access subject to approval at https://doi.org/10.17037/DATA.00003095, except for those from countries where data sharing is not permitted.

## APPENDIX 1

### National Vulnerable Newborn Collaborative Group for Stillbirths

**Argentina:** Veronica Pingray; Gabriela Gabriela; José Belizan; Luz Gibbons; Carlos Guevel.

**Australia:** Vicki Flenady; Adrienne Gordon; Kara Warrilow; Harriet Lawford.

**Denmark:** Erzsébet Horváth‐Puhó, Henrik T. Sørensen.

**Estonia:** Luule Sakkeus; Liili Abuladze.

**Lebanon:** Khalid A. Yunis; Ayah Al Bizri; Pascale Nakad.

**Malaysia:** Shamala Karalasingam; J. Ravichandran; R. Jeganathan; Nurakman Binti Baharum.

**Mexico:** Lorena Suárez‐Idueta; Arturo Barranco Flores; Jesus Felipe Gonzalez Roldan; Sonia Lopez Alvarez.

**The Netherlands:** Lisa Broeders; Aimée E. van Dijk.

**Qatar:** Fawzia Alyafei; Mai AlQubaisi; Tawa O. Olukade; Hamdy A. Ali; Mohamad Rami Alturk.

**Sweden:** Neda Razaz; Jonas Söderling.

**UK – England and Wales:** Lucy K. Smith; Bradley N. Manktelow; Ruth J. Matthews; Elizabeth Draper; Alan Fenton; Jennifer J. Kurinczuk.

**UK – Scotland:** Rachael Wood; Celina Davis; Kirsten Monteath; Samantha Clarke.

**Uruguay:** Isabel Pereyra, Gabriella Pravia.

**USA:** Sarka Lisonkova; Qi Wen.

**London School of Hygiene and Tropical Medicine:** Joy E. Lawn; Hannah Blencowe; Eric Ohuma; Yemi Okwaraji; Judith Yargawa; Ellen Bradley.

**Johns Hopkins University:** Robert Black; Joanne Katz; Dan Erchick; Elizabeth Hazel; Mike Diaz; Anne C. C. Lee.

## References

[1] World Health Organization . International classification of diseases eleventh revision (ICD‐11). Geneva: World Health Organization; 2022.

[2] UNICEF. United Nations Inter‐agency Group for Child Mortality Estimation (UN IGME) . Levels & trends in child mortality: report 2020. Estimates developed by the United Nations Inter‐Agency Group for Child Mortality Estimation. 2020.

[3] Heazell AEP , Siassakos D , Blencowe H , Burden C , Bhutta ZA , Cacciatore J , et al. Stillbirths: economic and psychosocial consequences. Lancet. 2016;387:604–616. 26794073 10.1016/S0140-6736(15)00836-3

[4] Lawn JE , Blencowe H , Waiswa P , Amouzou A , Mathers C , Hogan D , et al. Stillbirths: rates, risk factors, and acceleration towards 2030. Lancet. 2016;387:587–603. 26794078 10.1016/S0140-6736(15)00837-5

[5] Goldenberg RL , Saleem S , Goudar SS , Silver RM , Tikmani SS , Guruprasad G , et al. Preventable stillbirths in India and Pakistan: a prospective, observational study. BJOG. 2021;128(11):1762–1773. 34173998 10.1111/1471-0528.16820

[6] Lawn JE , Blencowe H , Oza S , You D , Lee ACC , Waiswa P , et al. Every newborn: progress, priorities, and potential beyond survival. Lancet. 2014;384:189–205. 24853593 10.1016/S0140-6736(14)60496-7

[7] World Health Organization . Every newborn: an action plan to end preventable deaths. Geneva: World Health Organization; 2014.

[8] Hug L , You D , Blencowe H , Mishra A , Wang Z , Fix MJ , et al. Global, regional, and national estimates and trends in stillbirths from 2000 to 2019: a systematic assessment. Lancet. 2021;398(10302):772–785. 34454675 10.1016/S0140-6736(21)01112-0 PMC8417352

[9] Poon LCY , Tan MY , Yerlikaya G , Syngelaki A , Nicolaides KH . Birth weight in live births and stillbirths. Ultrasound Obstet Gynecol. 2016;48(5):602–606. 27854393 10.1002/uog.17287

[10] Zhang X , Joseph KS , Cnattingius S , Kramer MS . Birth weight differences between preterm stillbirths and live births: analysis of population‐based studies from the U.S. and Sweden. BMC Pregnancy Childbirth. 2012;12:119. 23110432 10.1186/1471-2393-12-119 PMC3514233

[11] Flenady V , Koopmans L , Middleton P , Frøen JF , Smith GC , Gibbons K , et al. Major risk factors for stillbirth in high‐income countries: a systematic review and meta‐analysis. Lancet. 2011;377(9774):1331–1340. 21496916 10.1016/S0140-6736(10)62233-7

[12] Pels A , Beune IM , van Wassenaer‐Leemhuis AG , Limpens J , Ganzevoort W . Early‐onset fetal growth restriction: a systematic review on mortality and morbidity. Acta Obstet Gynecol Scand. 2020;99:153–166. 31376293 10.1111/aogs.13702 PMC7004054

[13] Ashorn P , Black RE , Lawn JE , Ashorn U , Klein N , Hofmeyr J , et al. The lancet small vulnerable newborn series: science for a healthy start. Lancet. 2020;396:743–745. 32919498 10.1016/S0140-6736(20)31906-1

[14] Lawn J , Ohuma E , Bradley E , Suárez‐Idueta L , Hazel E , Okwaraji Y , et al. Small babies, big risks: global estimates of prevalence and mortality for vulnerable newborns to accelerate change and improve counting. Lancet. 2023;401:1707–1719. 37167989 10.1016/S0140-6736(23)00522-6

[15] Suárez‐Idueta L , Blencowe H , Okwaraji Y , Yargawa J , Bradley E , Gordon A , et al. Neonatal mortality risk for vulnerable newborn types in 15 countries using 125.5 million nationwide linked birth outcome records, 2000 to 2020. BJOG. 2023;00:1–11. 10.1111/1471-0528.17506 PMC12678064 37156244

[16] Suárez‐Idueta L , Yargawa J , Blencowe H , Bradely E , Okwaraji Y . Vulnerable newborn types: analysis of population‐based registries for 165 million births in 23 countries, 2000 to 2021. BJOG. 2023;00:1–15. 10.1111/1471-0528.17505 PMC12678069 37156241

[17] Erchick DJ , Hazel EA , Katz J , Lee ACC , Diaz M , Wu LSF , et al. Vulnerable newborn types: analysis of subnational, population‐based birth cohorts for 541 285 live births in 23 countries, 2000–2021. BJOG. 2023;00:1–17. 10.1111/1471-0528.17510 PMC12678066 37156239

[18] Villar J , Ismail LC , Victora CG , Ohuma EO , Bertino E , Altman DG , et al. International standards for newborn weight, length, and head circumference by gestational age and sex: the newborn cross‐sectional study of the INTERGROWTH‐21st project. Lancet. 2014;384(9946):857–868. 25209487 10.1016/S0140-6736(14)60932-6

[19] Hazel EA , Erchick DJ , Katz J , Lee ACC , Diaz M , Wu LSF , et al. Neonatal mortality risk of vulnerable newborns: a descriptive analysis of subnational, population‐based birth cohorts for 238 143 live births in low‐ and middle‐income settings from 2000 to 2017. BJOG. 2023;00:1–12. 10.1111/1471-0528.17518 PMC12678067 37156238

[20] Brackett EE , Hall ES , Defranco EA , Rossi RM . Factors associated with occurrence of stillbirth before 32 weeks of gestation in a contemporary cohort. Am J Perinatol. 2022;39:84–91. 32736406 10.1055/s-0040-1714421

[21] McClure EM , Saleem S , Goudar SS , Garces A , Whitworth R , Esamai F , et al. Stillbirth 2010–2018: a prospective, population‐based, multi‐country study from the global network. Reprod Health. 2020;17:146. 33256783 10.1186/s12978-020-00991-y PMC7706249

[22] Agarwal U , Hugh O , Gardosi J . Prospective risk of stillbirth according to fetal size at term. J Perinat Med. 2022;50(9):1281–1282. 36205488 10.1515/jpm-2022-0449

[23] Hirst JE , Villar J , Victora CG , Papageorghiou AT , Finkton D , Barros FC , et al. The antepartum stillbirth syndrome: risk factors and pregnancy conditions identified from the INTERGROWTH‐21 st project. BJOG. 2018;125(9):1145–1153. 28029221 10.1111/1471-0528.14463 PMC6055673

[24] Melamed N , Baschat A , Yinon Y , Athanasiadis A , Mecacci F , Figueras F , et al. FIGO (International Federation of Gynecology and obstetrics) initiative on fetal growth: best practice advice for screening, diagnosis, and management of fetal growth restriction. Int J Gynecol Obstet. 2021;152(S1):152–157. 10.1002/ijgo.13522 PMC8252743 33740264

[25] Andreasen LA , Tabor A , Nørgaard LN , Rode L , Gerds TA , Tolsgaard MG . Detection of growth‐restricted fetuses during pregnancy is associated with fewer intrauterine deaths but increased adverse childhood outcomes: an observational study. BJOG. 2021;128(1):77–85. 32588532 10.1111/1471-0528.16380

[26] Vannevel V , Vogel JP , Pattinson RC , Adanu R , Charantimath U , Goudar SS , et al. Antenatal Doppler screening for fetuses at risk of adverse outcomes: a multicountry cohort study of the prevalence of abnormal resistance index in low‐risk pregnant women. BMJ Open. 2022;12(3):e053622. 10.1136/bmjopen-2021-053622 PMC8928296 35296477

[27] Ego A , Monier I , Skaare K , Zeitlin J . Antenatal detection of fetal growth restriction and risk of stillbirth: population‐based case–control study. Ultrasound Obstet Gynecol. 2020;55(5):613–620. 31364201 10.1002/uog.20414

[28] Carter EB , Stockburger J , Tuuli MG , Macones GA , Odibo AO , Trudell AS . Large‐for‐gestational age and stillbirth: is there a role for antenatal testing? Ultrasound Obstet Gynecol. 2019;54(3):334–337. 30353961 10.1002/uog.20162 PMC7543666

[29] Bukowski R , Hansen NI , Willinger M , Willinger M , Reddy UM , Parker CB , et al. Fetal growth and risk of stillbirth: a population‐based case‐control study. PLoS Med. 2014;11(4):e1001633. 24755550 10.1371/journal.pmed.1001633 PMC3995658

[30] Wood S , Tang S . Stillbirth and large for gestational age at birth. J Matern Fetal Neonatal Med. 2020;33(12):1974–1979. 30394187 10.1080/14767058.2018.1534229

[31] Liu S , Metcalfe A , León JA , Sauve R , Kramer MS , Joseph KS . Evaluation of the INTERGROWTH‐21st project newborn standard for use in Canada. PLoS One. 2017;12(3):e0172910. 28257473 10.1371/journal.pone.0172910 PMC5336248

[32] Man J , Hutchinson JC , Ashworth M , Heazell AE , Levine S , Sebire NJ . Effects of intrauterine retention and postmortem interval on body weight following intrauterine death: implications for assessment of fetal growth restriction at autopsy. Ultrasound Obstet Gynecol. 2016;48(5):574–578. 27781321 10.1002/uog.16018

[33] Yudkin PL , Wood L , Redman CWG . Risk of unexplained stillbirth AT different gestational ages. Lancet. 1987;329(8543):1192–1194. 10.1016/s0140-6736(87)92154-4 2883499

[34] Blondel B , Cuttini M , Hindori‐Mohangoo AD , Gissler M , Loghi M , Prunet C , et al. How do late terminations of pregnancy affect comparisons of stillbirth rates in Europe? Analyses of aggregated routine data from the Euro‐Peristat project. BJOG. 2018;125(2):226–234. 28557289 10.1111/1471-0528.14767

[35] Goldenberg RL , Nelson KG , Dyer RL , Wayne J . The variability of viability: the effect of physicians' perceptions of viability on the survival of very low‐birth weight infants. Am J Obstet Gynecol. 1982;143(6):678–684. 7091240 10.1016/0002-9378(82)90114-4

[36] Winyard A . The Nuffield Council on Bioethics Report — Critical care decisions in fetal and neonatal medicine: Ethical issues. Clinical Risk. 2007;13(2):70–73.

[37] Betrán AP , Temmerman M , Kingdon C , Mohiddin A , Opiyo N , Torloni MR , et al. Interventions to reduce unnecessary caesarean sections in healthy women and babies. Lancet. 2018;392:1358–1368. 30322586 10.1016/S0140-6736(18)31927-5

